# The Hygiene Hypothesis, Old Friends, and New Genes

**DOI:** 10.3389/fimmu.2019.00388

**Published:** 2019-03-06

**Authors:** John W. Frew

**Affiliations:** Laboratory of Investigative Dermatology, The Rockefeller University, New York, NY, United States

**Keywords:** autoimmunity, infection—immunology, genetics, allegy, monoclonal antibodies (immunology)

Allergic and autoimmune diseases such as asthma, psoriasis, rheumatoid arthritis, and inflammatory bowel disease vary in prevalence within human populations (1, 2). The hygiene hypothesis and more recently the “old friends” hypothesis have been quoted (3) to explain this disparity of prevalence, particularly between nations in the developed and developing world. Proposed etiological mechanisms include the absence of once-common childhood infections, as well as a reduction in exposure to a variety of commensal microorganisms in childhood (3). Exposure to such organisms is proposed to educate the immune system to appropriately respond to either innocuous and dangerous stimuli. Aberrant response to innocuous stimuli results in the development of allergic and autoimmune disease (3). However, these hypotheses alone do not fully account for the variability in prevalence of allergic and autoimmune disease (2, 4, 5). Certain populations exhibit an elevated risk to autoimmune and allergic disease above the background rate of individual human populations.

The advent of Genome Wide Association Studies (GWAS) have enabled identification of predisposing genetic variants to allergic and autoimmune disease (4, 5). They have also confirmed the association of previous loci identified in linkage studies. Genetic predisposition is a “*sine qua non”* for the development of allergic or autoimmune disease (4, 5) due to the ongoing evolutionary battle of protection against infectious disease whilst maintaining an acceptable risk of autoimmune disease which may impact upon reproductive capacity. In the identification of allergy and autoimmune disease-associated loci, multiple overlapping loci have been identified with pathogen-induced positive selection loci (6–12) (Table 1) (In this context, positive selection refers to genomic or Darwinian selection). A commonly known example of pathogen-induced positive selection is the increased prevalence of hemoglobin variants (HbS) causing sickle cell disease in populations with high previous malaria incidence, giving partial protection to severe malarial infection (10). In autoimmune and inflammatory disease, identified overlapping genes involve the activation and function of T cells, monocytes, NK cells, and dendritic cells as well as aspects of the major histocompatibility complex and transcription factors (13–17) (Table 1). Infectious agents associated with identified genes include *M. Leprae, M. Tuberculosis*, Y*. Pestis*, diarrheal illness, and *Plasmodium* sp. (4, 5, 8, 9, 11, 12) (Table 1). The presence of gene variants was associated with partial protection against the development of severe disease. Existing data is skewed by ascertainment bias with the majority of allergy and autoimmune GWAS have been undertaken in European populations. Also, GWAS for infectious disease require cohorts experiencing active infection, hence past endemic organisms with a high likelihood of producing positive selection (i.e., smallpox) are unable to be evaluated. Additionally, infectious agents with near saturation and multiple strains (i.e., *M. Tuberculosis*) indicate strong selective pressure (10), but due to the lack of comparison cohorts, positively selected genes are difficult to identify.

**Table 1:** List of identified genes identified in Autoimmune/Allergic Disease by GWAS overlapping with implicated pathogen-associated positive selection loci.

**Population**	**Candidate gene**	**Gene function**	**Allergic/autoimmune disease**	**Infection in which gene provides protective effect**	**References**
European	FUT2	Cell-cell interaction Cell-microbe interaction	Psoriasis Crohn's Disease	Viral diarrhoea	(5, 10, 11, 13)
European/African	TRIM65	Zinc ion binding Autophagy	Psoriasis	*Yersinia pestis*	(8)
European	HLA-DRB1	Major histocompatability complex	Atopic dermatitis	*Plasmodium falciparum* HBV persistance HCV Persistance	(4)
European	IFN-Gamma	Innate and adaptive immunity	Atopic dermatitis	*Mycobacterial* sp.	(11)
European	IL-12B	Inducer of Th1 immunity	Rheumatoid arthritis Multiple sclerosis	*M. Leprae*	(4, 10)
European	IL-21R	Proliferation of T, B, and NK Cells	Allergy (IgE Phenotype)	*M. Leprae*	(4, 5, 10)
European	IL-23R	Activation dendritic cells, monocytes, T cells and NK cells	Crohn's Ankylosing spondylitis	*Plasmodium* sp. *M. Leprae*	(4, 7)
European	TLR5	Pathogen recognition and innate immunity	Systemic lupus erythematosis	Salmonella	(4, 10)
European	TYK2	Innate and adaptive immune signaling	Rheumatoid arthritis Psoriasis Systemic lupus erythematosis Multiple sclerosis Type 1 diabetes	Protozoal infection	(4, 7, 10)
European	SNRPC	U1 small nuclear ribosome	Systemic lupus erythematosis	*M. Tuberculosis*	(10)
European	UHRF1BP1	Negative regulator of cell growth	Systemic lupus erythematosis	*M. Tuberculosis*	(4, 5, 10)
European/ African	IL12RB2	Inducer of Th1 immunity	Crohn's disease	*Plasmodium* sp.	(12)
Chinese	NOD2	Innate immune function	Crohn's disease	*M. Leprae*	(5, 10)
European	HLA-DQB1	Major histocompatability complex	Ulcerative colitis	*M. Leprae*	(4, 5, 7, 10)
European/ African	STAT4	Transcription factor T-cell maturation and function	Rheumatoid arthritis	Salmonella	(4, 7)
African Americans	APOL1	Serum apolipoprotein	SLE collapsing glomerulopathy	Tryptosomiasia	(10)

The corollary therefore is that for specific human population groups, historical exposure to infectious pathogens have positively selected for protective variants to improve survival and reproduction (6). One could hypothesize that, in the absence of infectious disease, these variants predispose to aberrant immune activation which, in the setting of appropriate environmental stimuli (such as a loss of “old friends” as well as smoking, metals, particulates, etc.) may manifest as allergy and autoimmune disease.

The implications of this correlation extend beyond population genetics to pharmacogenetics and emerging infectious diseases. The efficacy of monoclonal antibodies for control of autoimmune and allergic disease has associations with patient genetic variants (18) which have differing prevalence in various human populations. This could lead to targeted pharmacogenomic screening prior to treatment initiation. In the future this may become pertinent in emerging economies of East Asia [given the known high prevalence of NOD2 in Han Chinese, and the impact of NOD2 variants on therapy in Crohn's Disease (19)] and the Americas [in a similar vein to G6PD deficiency in Latin America (20)]. The re-emergence of vector-borne infectious diseases (such as malaria) secondary to climate change (21), may place individuals with autoimmune or allergic disease, who are being actively treated with immunomodulating therapies, at risk of infection. This risk may be greatest in those individuals where the therapy actively suppresses an inflammatory pathway known to be protective against the infectious agent. A relevant modern-day corollary is the risk of *M. Tuberculosis* reactivation during psoriasis treatment with TNF-alpha inhibitors in individuals of European ancestry [given the proposed mechanism of ancestral *Mycobacterium* sp. positively selecting for European psoriasis-associated genetic variants (22)]. This causal hypothesis requires validation in epidemiologic and functional studies.

In summary, the presence of overlapping gene associations identified by GWAS, as well as the evidence of pathogen-specific positive selection is an extension of the “hygiene/old friends” hypothesis which integrates findings from population genetics to explain disparate rates of autoimmune and allergic disease in different human populations. It also suggests avenues for further research in pharmacogenomics and susceptibility to emerging pathogens.

## Author Contributions

The author confirms being the sole contributor of this work and has approved it for publication.

### Conflict of Interest Statement

The author declares that the research was conducted in the absence of any commercial or financial relationships that could be construed as a potential conflict of interest.
